# Comprehensive characterization of the genetic landscape of familial Hirschsprung’s disease

**DOI:** 10.1007/s12519-023-00686-x

**Published:** 2023-03-01

**Authors:** Jun Xiao, Lu-Wen Hao, Jing Wang, Xiao-Si Yu, Jing-Yi You, Ze-Jian Li, Han-Dan Mao, Xin-Yao Meng, Jie-Xiong Feng

**Affiliations:** 1 Department of Pediatric Surgery, Tongji Hospital, Tongji Medical College, Huazhong University of Science and Technology, 1095 Jiefang Avenue, Wuhan, 430030 China; 2 Hubei Clinical Center of Hirschsprung’s Disease and Allied Disorders, Wuhan, 430030 China; 3 Department of Radiology, Tongji Hospital, Tongji Medical College, Huazhong University of Science and Technology, 1095 Jiefang Avenue, Wuhan, 430030 China

**Keywords:** Genetic characteristics, Hirschsprung’s disease, Penetrance, Recurrence risk, Ret proto-oncogene (*RET*)

## Abstract

**Background:**

Hirschsprung’s disease (HSCR) is one of the most common congenital digestive tract malformations and can cause stubborn constipation or gastrointestinal obstruction after birth, causing great physical and mental pain to patients and their families. Studies have shown that more than 20 genes are involved in HSCR, and most cases of HSCR are sporadic. However, the overall rate of familial recurrence in 4331 cases of HSCR is about 7.6%. Furthermore, familial HSCR patients show incomplete dominance. We still do not know the penetrance and genetic characteristics of these known risk genes due to the rarity of HSCR families.

**Methods:**

To find published references, we used the title/abstract terms “Hirschsprung” and “familial” in the PubMed database and the MeSH terms “Hirschsprung” and “familial” in Web of Science. Finally, we summarized 129 HSCR families over the last 40 years.

**Results:**

The male-to-female ratio and the percentage of short segment-HSCR in familial HSCR are much lower than in sporadic HSCR. The primary gene factors in the syndromic families are ret proto-oncogene (*RET*) and endothelin B receptor gene (*EDNRB*). Most families show incomplete dominance and are relevant to *RET*, and the *RET* mutation has 56% penetrance in familial HSCR. When one of the parents is a *RET* mutation carrier in an HSCR family, the offspring’s recurrence risk is 28%, and the incidence of the offspring does not depend on whether the parent suffers from HSCR.

**Conclusion:**

Our findings will help HSCR patients obtain better genetic counseling, calculate the risk of recurrence, and provide new insights for future pedigree studies.

**Supplementary Information:**

The online version contains supplementary material available at 10.1007/s12519-023-00686-x.

## Introduction

Hirschsprung’s disease (HSCR) is one of the most common congenital digestive tract malformations, with an incidence of 1 in 5000 and a male‒female ratio of 4:1. Due to the loss of enteric neurons in the distal colon, HSCR can cause stubborn constipation or gastrointestinal obstruction after birth due to loss of enteric neurons in the distal colon, bringing great physical and mental pain to patients and their families [1]. With the development of sequencing technology and bioinformatics analysis, more than 20 genes [ret proto-oncogene (*RET*), endothelin B receptor gene (*EDNRB*), paired-like homeobox 2B gene (*PHOX2B*), etc.] have been linked to HSCR [2–5].

Although most cases of HSCR are sporadic, some families with two or more HSCR family members are classified as HSCR families. Three studies reported a series of HSCR families [6–8], implying that HSCR has a genetic predisposition. Mc Laughlin and Puri reported a 7.6% overall rate of familial recurrence in 4331 HSCR index cases [9]. Familial HSCR is also due to a list of pathogenic genes, such as *RET* [10], *EDNRB* [11], and *PHOX2B* [12]. However, familial HSCR does not follow Mendelian inheritance, and *RET* and *PHOX2B* show incomplete penetrance in members of familial HSCR [10, 12]. Because HSCR families are uncommon, current gene studies are based on a summary of a few families. The penetrance and genetic characteristics of these known risk genes in familial HSCR, particularly the major pathogenic gene *RET*, which occurs in 50% of familial HSCR and 35% of sporadic HSCR, remain unknown [13].

In this study, we summarized 129 HSCR families reported in 53 references to analyze the penetrance, recurrence risk, and genetic characteristics of familial HSCR. Our study will elucidate the genetic characteristics of familial HSCR, provide preferable genetic counseling for HSCR patients, help in calculating the risk of recurrence, and provide new insights for future pedigree studies.

## Methods

We used the title/abstract terms “Hirschsprung” and “familial” in the PubMed database and the MeSH terms “Hirschsprung” and “familial” in the Web of Science to search published references. At least two family members with HSCR were required for inclusion. Studies of multicenter investigated data were excluded to avoid including duplicate families in the analysis. Finally, we confirmed and analyzed 53 references containing 129 families (Supplemental Table 1) [6–8, 10–12, 14–60].

The group-divisible designs are as follows: (1) HSCR subtypes: short segment-HSCR (S-HSCR), long segment-HSCR (L-HSCR), total colonic aganglionosis (TCA), total bowel aganglionosis, not available (NA); (2) syndromic HSCR and non-syndromic HSCR/not mentioned, syndromic HSCR refers to HSCR patients suffering other syndromic symptoms, such as familial medullary thyroid cancer, multiple endocrine neoplasia type 2a (MEN2A) and Waardenburg syndrome; (3) male and female; (4) familial genetic characteristics: “parent to child, lineal 3 generation” means determined HSCR occurs in both the proband’s parents (father, mother, or both) and grandparents (grandfather, grandmother, or both), “parent to child, lineal 2 generation” means determined HSCR occurs in the proband’s parents (father, mother, or both), “siblings” means determined HSCR occurs in the proband’s brothers or sisters, “collateral relatives” means determined HSCR occurs in the proband’s cousins or relatives; (5) syndromic symptoms: familial medullary thyroid cancer (FMTC), MEN2A, Waardenburg syndrome, Bardet–Biedl syndrome, special physical characteristics, respiratory symptoms, external auditory canal agenesis, anisocoria, multiple sclerosis, congenital central hypoventilation syndrome, Currarino syndrome, neuroblastoma, congenital heart disease, trisomy 21, meningocele and intellectual disability; (6) genetic patterns or pathogenic mechanisms: dominant inheritance, recessive inheritance, incomplete dominance, compound heterozygous inheritance, epistasis; (7) transmission pattern: affected father to affected son, affected father to unaffected son, affected father to affected daughter, affected father to unaffected daughter, unaffected father to affected son, unaffected father to unaffected son, unaffected father to affected daughter, unaffected father to unaffected daughter, affected mother to affected son, affected mother to unaffected son, affected mother to affected daughter, affected mother to unaffected daughter, unaffected mother to affected son, unaffected mother to unaffected son, unaffected mother to affected daughter, unaffected mother to unaffected daughter (Supplementary Table 2).

We analyzed the penetrance and transmission patterns of the *RET* gene. Inclusion criteria are as follows: (1) families with equal to or more than two members diagnosed with HSCR; (2) the *RET* mutation was linked to families; (3) detailed *RET* mutation sites are available; and (4) there is only one variation of *RET*. The exclusion criteria were as follows: (1) consanguineous marriage; (2) unavailable *RET* mutation information or de novo variants; and (3) equal to or more than two variations of *RET*. Finally, we summarized 110 *RET* carriers from 21 references (Supplementary Table 3) [10, 16–19, 21, 24, 27, 30, 31, 36–40, 43, 44, 47, 50, 52, 54].

## Results

### Sex ratio of HSCR subtypes and syndromic HSCR in familial HSCR

**Fig. 1:**
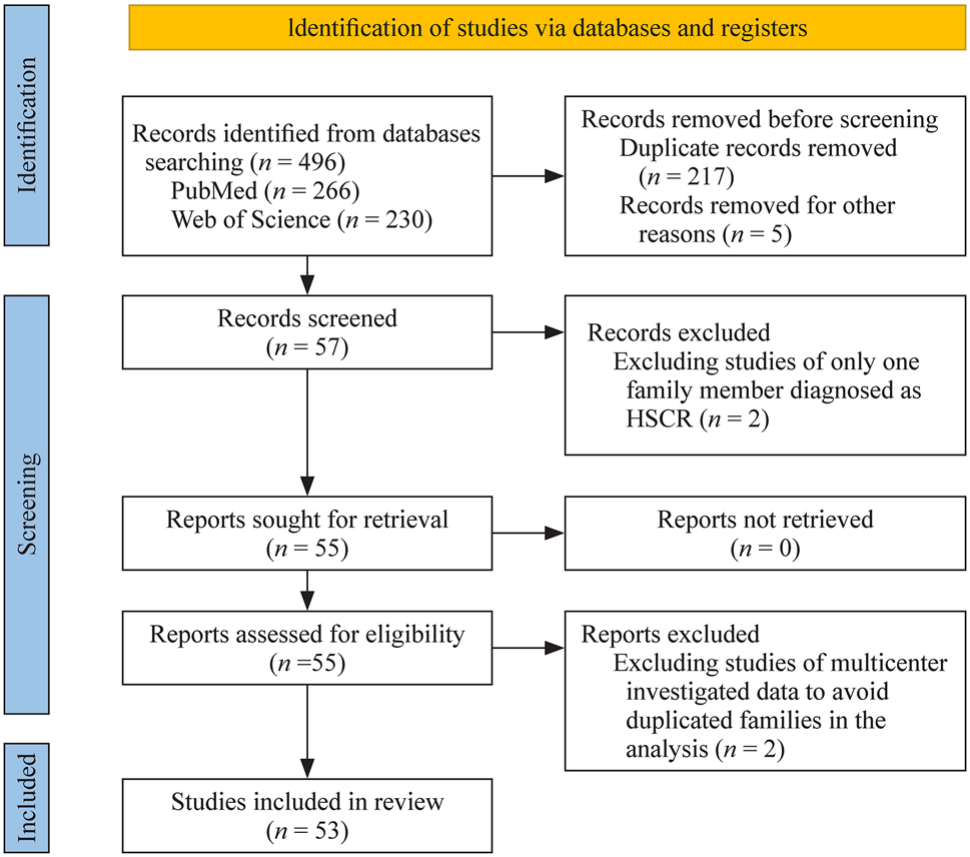
Preferred Reporting Items for Systematic Review and Meta-Analysis 2020 flow diagram for new systematic reviews that included searches of databases and registers only. *HSCR* Hirschsprung’s Disease

We finally discovered 53 references (Fig. 1), including 129 families with 416 HSCR cases (Tables 1 and 2). The ratio of males to females in S-HSCR was 1.11 (39/35); the ratio of males to females in L-HSCR was 1.48 (43/29); the ratio of males to females in TCA/total bowel aganglionosis was 0.78 (7/9); and the ratio of males to females in all familial HSCR cases was 1.51 (250/166) (Table 1), which is much lower than the sporadic HSCR ratio (4:1) [61]. The male-to-female ratio in syndromic HSCR was 1.11 (39/35), and the male-to-female ratio in non-syndromic HSCR/not mentioned was 1.61 (211/131) (Table 2). The percentages of S-HSCR, L-HSCR, TCA and total bowel ananglionos in familial HSCR cases are 46% (74/162), 44% (72/162), 8% (13/162) and 2% (3/162), respectively, while the reported percentage of S-HSCR reported in sporadic HSCR cases is 80% [61].

**Table 1:** Analysis between sex ratio and HSCR subtypes

Variables	S-HSCR	L-HSCR	TCA	Total bowel aganglionosis	NA	Total
Male	39	43	7	0	161	250
Female	35	29	6	3	93	166
Total	74	72	13	3	254	416

*HSCR* Hirschsprung’s disease, *S-HSCR* short segment-HSCR, *L-HSCR* long segment-HSCR, *TCA* total colonic aganglionosis, *NA* not available

**Table 2:** Analysis between sex ratio, syndromic HSCR and non-syndromic HSCR

Variables	Syndromic HSCR	Non-syndromic HSCR or not mentioned	Total
Male	39	211	250
Female	35	131	166
Total	74	342	416

“Syndromic symptoms” means there are other concomitant diseases besides HSCR, such as Waardenburg syndrome and familial medullary thyroid cancer. *HSCR* Hirschsprung’s disease

### Syndromic HSCR families exist mainly in sibling families

We examined the 129 families (Table 3). Syndromic HSCR families accounted for 30% (39/129); parent to child (lineal 2 generation) families accounted for 32% (41/129); and sibling families accounted for 53% (68/129). The ratio of syndromic HSCR families to non-syndromic/not mentioned HSCR families in sibling families is 0.48 (22/46); in parent to child (lineal 2 generation) families, the ratio is 0.28 (9/32); and in all families, the ratio of syndromic HSCR families to non-syndromic/not mentioned HSCR families is 0.43 (39/90). Sibling families account for the majority of syndromic HSCR families (22/39, 56%); sibling and parent to child (lineal 2 generation) families are the most common familial HSCR characteristics.

**Table 3:** Analysis between family characteristics, syndromic Hirschsprung’s disease (HSCR) and non-syndromic HSCR

Family characteristics	Syndromic HSCR	Non-syndromic HSCR or not mentioned	Total	Percentage (%)
Parent to child				
Lineal 3 generation^a^	3	3	6	4.65
Lineal 2 generation^b^	9	32	41	31.78
Siblings^c^ (including twins)	22	46	68	52.71
Collateral relatives^d^	5	9	14	10.85
Total	39	90	129	
Percentage (%)	30.23	69.77		

When a large family exists multiple patterns, we choose the priority order refer to “parents to child > siblings > collateral relatives”. ^a^Determined HSCR occurs in both proband’s parents (father, mother or both) and grandparents (grandfather, grandmother or both); ^b^determined HSCR occurs in proband’s parents (father, mother or both); ^c^determined HSCR occurs in proband’s brothers or sisters; ^d^determined HSCR occurs in proband’s cousins or relatives

### The primary genetic factors of syndromic familial HSCR are *RET* and *EDNRB*

There were 74 HSCR patients with syndromic symptoms in 39 families (Table 4). FMTC, MEN2A, and Waardenburg syndrome families accounted for 41% (16/39) and 31% (12/39), respectively, of the total; FMTC/MEN2A and Waardenburg syndrome patients accounted for 39% (29/74) and 31% (29/73) of the total, respectively. FMTC/MEN2A, Waardenburg syndrome, and intellectual disability are all linked to *RET* mutations; *EDNRB* mutations are linked to Waardenburg syndrome, special physical characteristics, and multiple sclerosis; and *PHOX2B* mutations can cause respiratory symptoms, anisocoria, congenital central hypoventilation syndrome, and congenital heart disease.

**Table 4:** Analysis between syndromic symptoms and risk genes

Syndromic symptoms	Families	Cases	Gene association
FMTC/MEN2A	16	29	*RET*
Waardenburg syndrome	12	22	*EDNRB/EDN3, RET/PAX3, ERBB3*
Bardet–Biedl syndrome	3	6	–
Special physical characteristics	1	3	*EDNRB*
Respiratory symptoms	1	3	*PHOX2B*
External auditory canal agenesis	1	2	*ERBB3*
Anisocoria	1	2	*PHOX2B*
Multiple sclerosis	1	2	*EDNRB*
Congenital central hypoventilation syndrome	1	2	*PHOX2B*
Currarino syndrome	1	2	–
Neuroblastoma	1	2	–
Congenital heart disease	1	1	*PHOX2B*
Trisomy 21	1	1	–
Meningocele	1	1	–
Intellectual disability	1	1	*RET/BBS*

Special physical characteristics include telecanthus, prominent nasal bridge, broad nasal bridge, tapering fingers, widely spaced nipples, low set ears, short neck, thoracic scoliosis, mild syndactyly and broad hallux. *FMTC* familial medullary thyroid cancer, *MEN2A* multiple endocrine neoplasia type 2a, *RET* ret proto-oncogene, *EDNRB* endothelin B receptor gene, *EDN3* endothelin 3 gene, *PAX3* paired box 3 gene, *ERBB3* v-erb-b2 erythroblastic leukemia viral oncogene homolog 3 gene, *PHOX2B* paired like homeobox 2B gene, *BBS* Bardet–Biedl gene. “–” no data

### Familial HSCR has complicated genetic patterns

There were 62 families with detailed gene information in this analysis (Table 5). Twenty-seven percent (17/62) of families show dominant inheritance, and 47% (29/62) of families show incomplete dominance. *RET*-associated families accounted for 65% (40/62). In 40 *RET*-associated families, 30% (12/40) showed dominant inheritance, and 58% (23/40) showed incomplete dominance.

**Table 5:** Analysis between pattern of gene role in families and risk genes

Pattern of gene role in families	Dominant inheritance	Recessive inheritance	Incomplete dominance	Compound heterozygous inheritance	Epistasis	All
*RET*	12	0	23	0	0	35
*RAT/PAX3*	0	0	0	0	1	1
*RET/NTRK3*	0	0	0	0	1	1
*RET/LRBA/GLI3*	0	0	0	0	1	1
*RET/BBS*	0	0	0	0	2	2
*EDBNB*	1	1	1	0	0	3
*EDN3*	0	1	0	0	0	1
*EDN3/EDNRB*	0	0	0	0	1	1
*ERBB3*	0	1	0	0	0	1
*GDNF*	0	0	1	0	0	1
*GFRA1/ZHX2/TPCNI*	0	0	0	0	1	1
*PHOX2B*	1	0	2	0	0	3
*CYP2B6*	0	0	1	0	0	1
*NRG1/SEMA3C*	0	0	1	0	0	1
*NTRK1*	1	0	0	0	0	1
*PCDHA9*	0	1	0	0	0	1
*DPYD*	1	0	0	0	0	1
*PLAU/FBN1*	0	0	0	0	1	1
*NTF3/IRAK3/KDR*	0	0	0	0	1	1
*CNTN5*	1	0	0	0	0	1
*CREBBP/TSC2*	0	0	0	0	1	1
*FAT3/SEMA3D/PTCH1*	0	0	0	0	1	1
*AHNAK*	0	0	0	1	0	1
All	17	4	29	1	11	62

*RET* ret proto-oncogene, *PAX3* paired box 3 gene, *NTRK3* neurotrophic tyrosine receptor kinase 3 gene, *LRBA* lipopolysaccharide-responsive beige-like anchor protein gene, *GLI3* GLI-Kruppel family member 3 gene, *BBS* Bardet–Biedl gene, *EDNRB* endothelin B receptor gene, *EDN3* endothelin 3 gene, *ERBB3* v-erb-b2 erythroblastic leukemia viral oncogene homolog 3 gene, *GDNF* glial cell line-derived neurotrophic factor gene, *GFRA1* GDNF family receptor alpha-1 gene, *ZHX2* zinc fingers and homeoboxes 2 gene, *PHOX2B* paired-like homeobox 2B gene, *CYP2B6* cytochrome P450 2B6 gene, *NRG1* neuregulin-1 gene, *SEMA3C* semaphorin 3C gene, *PCDHA9* protocadherin alpha 9 gene, *DPYD* dihydropyrimidine dehydrogenase gene, *PLAU* plasminogen activator urokinase gene, *FBN1* fibrillin-1 gene, *NTF3* neurotrophin-3 gene, *IRAK3* interleukin-1 receptor-associated kinase 3 gene, *KDR* kinase-insert domain-containing receptor gene, *CNTN5* contactin-5 gene, *CREBBP* CREB-binding protein gene, *TSC2* tuberous sclerosis 2 gene, *FAT3* FAT atypical cadherin 3 gene, *SEMA3D* semaphorin 3D gene, *PTCH1* patched 1 gene

### The penetrance of the *RET* mutation and recurrence risk in familial HSCR

**Fig. 2:**
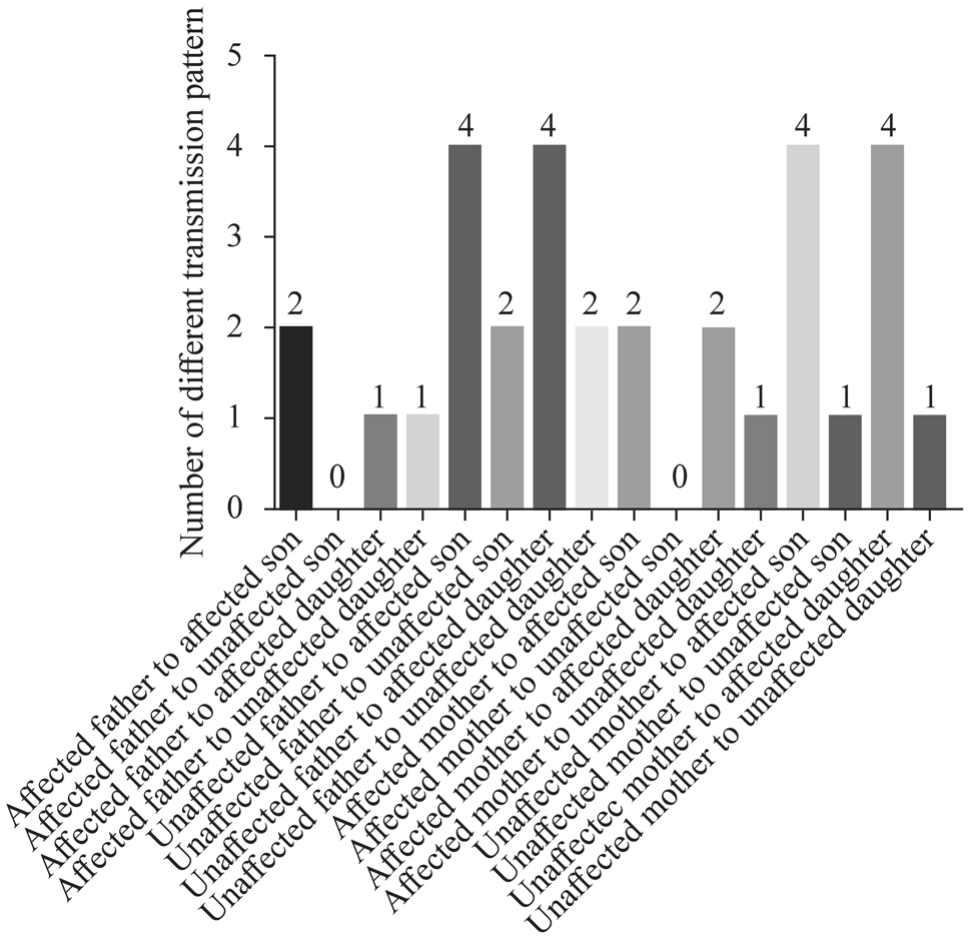
Number of different transmission patterns in *RET* mutation carriers. *RET* ret proto-oncogene

There are 110 RET mutation carriers in 21 familial HSCR references. The number of affected carriers (HSCR and the *RET* mutation) is 62, implying that the *RET* mutation is 56% (62/110) penetrant in familial HSCR (“carriers” means *RET* gene mutation members; “affected carriers” means HSCR along with *RET* mutation). As a result, when one of the parents is a *RET* mutation carrier in an HSCR family, the offspring’s recurrence risk is 28% (1/2 of 56%). We counted the number of different transmission patterns (Fig. 2): unaffected *RET*-carrying parents transmitted the variation to children, and the percentage of affected children was 73% (16/22); affected *RET*-carrying parents transmitted the variation to children, and the percentage of affected children was 78% (7/9). They seem to have a similar HSCR rate, meaning that the incidence of HSCR in offspring does not depend on whether the parents suffer from it.

## Discussion

Previous studies only looked at a few HSCR families due to their rarity. They usually focus on one of these pathogenic genes and study its biological function. However, there is a lack of systematic research on the genetic characteristics and gene penetrance of familial HSCR. We summarized all 129 families reported in this study over the last 40 years, concluded that the genetic feature of familial HSCR exists, provided preferable genetic counseling for HSCR patients, assisted in calculating the risk of recurrence, and provided new insights for future pedigree studies.

We determined that 65% of HSCR families were associated with *RET* in our study. *RET* shows complex genetic patterns, including dominant inheritance (30%) and incomplete dominance (58%). We determined the penetrance of the *RET* mutation to be 56% in familial HSCR. That is, when a member of a *RET*-associated family is diagnosed with HSCR, other carriers with the *RET* mutation have a 56% chance of developing HSCR. When one of the parents is a *RET* mutation carrier in an HSCR family, the offspring’s recurrence risk is 28%.

A few risk genes are highly associated with familial HSCR and show multiple genetic characteristics. The function of *RET* in HSCR is complicated and varied. Previous research has revealed the dominant inheritance and incomplete dominance of *RET*. However, some scholars have also proposed dosage-dependent penetrance and epistasis to explain the phenomenon of incomplete dominance and different subtypes of HSCR [30, 33]. For other risk genes, *EDNRB* shows dominant inheritance, recessive inheritance and incomplete dominance, and *PHOX2B* shows dominant inheritance and incomplete dominance. Thirty percent of families are syndromic, and 56% (22/39) of syndromic HSCR families are sibling groups. Syndromic symptoms (FMTC/MEN2A and Waardenburg syndrome) are mainly caused by *RET* and *EDNRB*. Syndrome symptoms are common and varied, so it is important to focus on other complications in HSCR patients and pay attention to risk genes, such as *RET*, *EDNRB* and *PHOX2B*.

Twelve percent (15/129) of the families had consanguineous marriages. Consanguineous marriage appears to be a risk factor for HSCR, and it is recommended that consanguineous marriage be avoided. We also observed 11 pairs of twins (six pairs of monozygotic twins and five pairs of dizygotic twins). In six pairs of monozygotic twins, four pairs (six males and two females) were diagnosed with HSCR, while there was only one case diagnosed with HSCR in the remaining two pairs. Two pairs of dizygotic twins were diagnosed with HSCR, while the remaining pairs had only one HSCR patient. Thus, it seems that the risk of HSCR was independent in both monozygotic and dizygotic twins due to the incomplete dominance of HSCR.

The mutated genes or loci, especially the gene *RET*, reported in the families in the references we included conform to the law of genetic coseparation and are predicted or proven to be highly pathogenic. Based on this, all reported mutated genes or loci were included and analyzed. We performed a large familial HSCR study and determined a series of ratios and percentages. However, there may be some statistical bias, as the reported HSCR families are typical and characteristic.

In conclusion, the male-to-female ratio in familial HSCR is close to one. Most families show incomplete dominance and are relevant to *RET*, and the *RET* mutation has 56% penetrance in familial HSCR. The incidence of HSCR in the offspring does not depend on whether the parent suffers from HSCR. Overall, our findings will enhance the comprehensive characterization of the genetic landscape for familial HSCR and help HSCR patients obtain better genetic counseling.

## Supplementary Information

Below is the link to the electronic supplementary material.

Supplementary file 1 (XLSX 42 KB)

## Data Availability

All data generated or analyzed during this study are included in this published article (and its supplementary information files).
